# Comparative Genomic Analysis of Multidrug-Resistant *Pseudomonas aeruginosa* Clinical Isolates VRFPA06 and VRFPA08 with VRFPA07

**DOI:** 10.1128/genomeA.00140-14

**Published:** 2014-03-06

**Authors:** Nandagopal Murugan, Jambulingam Malathi, Vetrivel Umashankar, Hajib Narahari Rao Madhavan

**Affiliations:** a Dept. of Microbiology, L & T Microbiology Research Centre, Vision Research Foundation, Sankara Nethralaya, Chennai, Tamil Nadu, India; b Centre for Bioinformatics, Vision Research Foundation, Sankara Nethralaya, Chennai, Tamil Nadu, India; c SASTRA University, Thanjavur, Tamil Nadu, India

## Abstract

*Pseudomonas aeruginosa* isolates harboring acquired drug-resistant genes lead to increased mortality. Here, we have sequenced and annotated the genomes of two multidrug-resistant (MDR) *P. aeruginosa* isolates and a susceptible *P. aeruginosa* clinical isolate evidencing divergent antibiotic susceptibilities. Genomic analysis showed insight on the different genomic strategies adapted by *P. aeruginosa* to combat antimicrobial effects.

## GENOME ANNOUNCEMENT

*Pseudomonas aeruginosa* causes urinary tract infections, bacteremia, gastrointestinal infections, and a variety of systemic infections. Multidrug-resistant (MDR) *P. aeruginosa*-mediated infections can lead to serious outcomes, such as amputation, or in the worst case, death (1). The intrinsic and acquired resistance mechanisms of *P. aeruginosa* include the production of β-lactamases, efflux pumps, and target site or outer membrane modifications.

The *P. aeruginosa* strains VRFPA06 (isolated from blood), VRFPA07 (from a rectal swab), and VRFPA08 (from a urine specimen) were submitted to the L & T Microbiology Research Centre, Sankara Nethralaya, Chennai, Tamil Nadu, India, and studied. The antimicrobial susceptibility result showed that VRFPA06 and VRFPA08 are resistant to aminoglycosides, fluoroquinolones, and β-lactams up to carbapenem but were susceptible to the monobactam aztreonam. VRFPA07 is susceptible to all commonly used drugs.

Hence, we determined the draft genome sequences of two MDR *P. aeruginosa* strains, VRFPA06 and VRFPA08, and one sensitive strain, VRFPA07, to decipher the differences between susceptible and resistant strains at a genomic level, in order to understand the highly combative phenotype of the species. The whole-genome sequencing was performed using the Ion Torrent PGM sequencer with 400-bp read chemistry (Life Technologies). The sequencing protocol was performed according to a previous study (2). The filtered sequences were made into a reference-guided assembly against the whole-genome sequence of *P. aeruginosa* PA14 and B136-33 using CLC Genomics Workbench software version 6.5 (CLC bio, Germantown, MD). The assembled data were subjected to RAST annotation (3) and the Prokaryotic Genomes Automatic Annotation Pipeline (PGAAP) (http://www.ncbi.nlm.nih.gov/genomes/static/Pipeline.html). The sequencing and annotation results are given in Table 1.

**TABLE 1:** Sequencing and annotation results of VRFPA06, VRFPA07, and VRFPA08

Strain features	VRFPA06	VRFPA07	VRFPA08
NCBI accession no.	AYSK00000000.1	AZBO00000000.1	AZHU00000000.1
Genome coverage (×)	100	80	70
Genome size (bp)	6,975,032	7,177,216	7,035,331
No. of contigs	276	140	197
Isolation source	Blood	Rectal swab	Urine
G+C content (%)	66.10	65.90	66.10
No. of genes	6,650	6,916	6,794
No. of pseudogenes	288	84	51
No. of proteins	6,277	6,764	6,661
No. of rRNAs	16 (5S, 16S, 23S)	9 (5S, 16S, 23S)	14 (5S, 16S, 23S)
No. of tRNAs	66	57	64
No. of ncRNAs	3	1	4

The identities of the strains were confirmed by *in silico* multilocus sequence typing (MLST) (http://cge.cbs.dtu.dk/services/) using the *P. aeruginosa* MLST database targeting seven potential loci (*acsA*, *aroE*, *guaA*, *mutL*, *nuoD*, *ppsA*, and *trpE*) and defined as VRFPA06 (sequence type 664 [ST-664]), VRFPA07 (ST-313), and VRFPA08 (ST-823) (4). Further analysis was carried out using the Web server ResFinder (4), an *in silico* tool used to decipher acquired antimicrobial resistance genes among draft genomes. Drug resistance genes, namely, aminoglycoside [*aph*(*3*′)-*IIb*], fosfomycin (*fosA*), and β-lactam (*bla*_OXA-50_ and *bla*_PAO_) genes, were detected among all three isolates of the VRFPA06, VRFPA07, and VRFPA08 strains. Other resistance genes observed among the VRFPA06 and VRFPA08 strains were the sulfonamide resistance Sul1 gene, the chloramphenicol resistance CatB7 gene, and the tetracycline resistance TetG gene. The β-lactam resistance gene *bla*_OXA-40_ and the fluoroquinolone [*aac(6′)-Ib-cr*] gene in VRFPA06 and the metallo-β-lactamase (MBL) gene *bla*_VIM-2_ and the trimethoprim resistance *dfrB5* gene in VRFPA08 were uniquely detected.

The preliminary genomic analysis in our study predicted the presence of *bla*_VIM-2_, an MBL gene located on different class 1 integrons of the VRFPA08 strain. The OXA-type carbapenemase gene *bla*_OXA-40_ in VRFPA06 might be responsible for broad-spectrum resistance to β-lactam drugs. The ability to resist aminoglycosides is mediated by the *aph(3′)-IIb*, *aac(6′)-Ib-cr*, and *aac(6′)-Ib-cr* genes in VRFPA06 and the *aac(3)-Id* and *aph(3′)-IIb* genes in VRFPA08. In spite of the genome sizes being similar, the numbers of RNA-coding genes were higher in VRFPA06 (85) and VRFPA08 (82) than in VRFPA07, which has only 67 RNA-coding genes, resulting in a higher expression of proteins among drug-resistant strains than in susceptible ones. Further deep analyses will provide better insights on other mechanisms involved in this strains.

### Nucleotide sequence accession numbers.

This whole-genome shotgun project of *P. aeruginosa* strains VRFPA06, VRFPA07, and VRFPA08 has been deposited at DDBJ/EMBL/GenBank under accession no. AYSK00000000, AZBO00000000, and AZHU00000000, respectively. The versions described in this paper are the first versions, AYSK00000000.1, AZBO00000000.1, and AZHU00000000.1, respectively.
